# Identifying Strengths and Gaps in Data Management and Reporting through Malaria Routine Data Quality Assessment: Results from Two Health Regions in Madagascar

**DOI:** 10.4269/ajtmh.23-0224

**Published:** 2024-01-23

**Authors:** Maurice Yé, Jean Marie N’Gbichi, Tokinirina Andrianantoandro, Sabas Rabesahala, Urbain Rabibizaka, Brune Estelle Ramiranirina, Omega Rabeola, Laurent Kapesa, Yazoumé Yé

**Affiliations:** ^1^ U.S. President’s Malaria Initiative Measure Malaria, University of North Carolina, Chapel Hill, North Carolina/ICF, Rockville, Maryland;; ^2^ National Malaria Control Program, Antananarivo, Madagascar;; ^3^ U.S. President’s Malaria Initiative, Antananarivo, Madagascar

## Abstract

In 2021, the U.S. President’s Malaria Initiative Measure Malaria project provided support to the National Malaria Program to conduct a data quality assessment. The main goal was to help health centers enhance the quality of their malaria data. The assessment involved reviewing data from outpatient registers, monthly reports, and DHIS2 data. Reporting timeliness, completeness, data element completeness, and availability of source documents were assessed. For timeliness, the assessment measured the proportion of reports that were submitted on time out of the expected total. The results showed that the reporting timeliness was inadequate in Atsinanana (85%) and adequate for Atsimo-Andrefana (95%). Data elements completeness, which refers to reports without missing data, was inadequate in Atsinanana (43%) and Atsimo-Andrefana (68%). The availability of source documents, such as records forms, was assessed and found to be 59% in Atsimo-Andrefana and 48% in Atsinanana. The use of standard reporting forms, which ensures consistency and accuracy in reporting, was reported to be 44% in Atsinanana and 54% in Atsimo-Andrefana. Data discrepancies were identified between outpatient registers, monthly reports, and DHIS2 data. A verification factor (VF) was used to compare the figures in these different sources. The VF was 1.2 in Atsinanana and 1.1 in Atsimo-Andrefana for both monthly reports and DHIS2 data, indicating an overreporting of fever cases tested in 6- to 13-year-olds. Overall, the assessment revealed gaps in data elements completeness, reporting accuracy, and availability of data recording guidelines. The findings suggest that regular data quality assessments should be implemented to guide decision making in Madagascar.

## INTRODUCTION

In Madagascar, perceived low quality of routine data limits their use for decision-making. To help the National Malaria Program (NMCP) address this challenge, MEASURE Evaluation project, funded by the U.S. Agency for International Development and the U.S. President’s Malaria Initiative (PMI) developed a standard malaria routine data quality assessment tool^1^ (MRDQA) adapted from the WHO data quality review supervisory checklist.^2^

The MRDQA tool^1^ is specifically designed for assessing routine malaria data quality during regular supportive supervision visits. It consists of three main components: checking the quality of the data, evaluating the performance system that produces the data, and creating action plans to enhance both the health information system functions and the quality of the data. By using the MRDQA tool, the malaria surveillance systems can be strengthened in accordance with WHO recommendations,^3^^,^^4^ leading to improved data quality at service delivery points. Moreover, this tool helps standardize data quality assessments across health facilities and enables comparison and measurement of progress over time. It has already been adopted and implemented in several countries such as Cote d’Ivoire, the Democratic Republic of the Congo (DRC), Kenya, Mali, Niger, and Sierra Leone.^5^

In Madagascar, the MRDQA tool was introduced as a pilot initiative to assess and monitor malaria data quality, as well as to enhance data utilization for informing malaria interventions in selected regions. This article presents the results of the pilot phase, with the objective of conducting data quality assessments to improve program monitoring, decision-making, and overall contributions to strengthening the health management information system (HMIS).

## MATERIALS AND METHODS

### Study design.

In 2021, the MRDQA (Malaria Routine Data Quality Assessment) was conducted in Madagascar’s Atsinanana and Antsimo-Andrefana health regions. These regions were selected as representative of moderate (50 to 100 cases per 1,000 inhabitants) for Atsinanana and high (over 100 cases per 1,000 inhabitants) for Atsimo-Andrefana (NMP strategic plan 2018–2021 epidemiological stratification).

Convenience sampling was used to choose health centers for the assessment, prioritizing geographic accessibility and data availability. Health centers with a reporting timeliness rate of <70% in 2021 were included. Reporting timeliness defined as annual average of monthly reports submitted before the 15th of the following month. In Atsinanana, 14 out of 52 health centers were selected, and in Antsimo-Andrefana, 16 out of 65 health centers were included in the assessment.

### Study sites.

**Figure 1.**
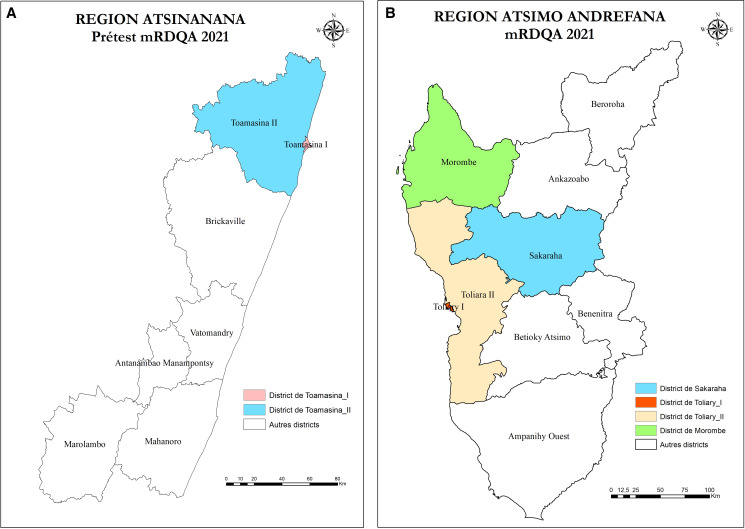
Location of study health centers. (**A**) Atsinanana. (**B**) Antsimo-Andrefana.

Figure 1 shows the location of study health centers.

### Description of the MRDQA tool.

**Figure 2.**
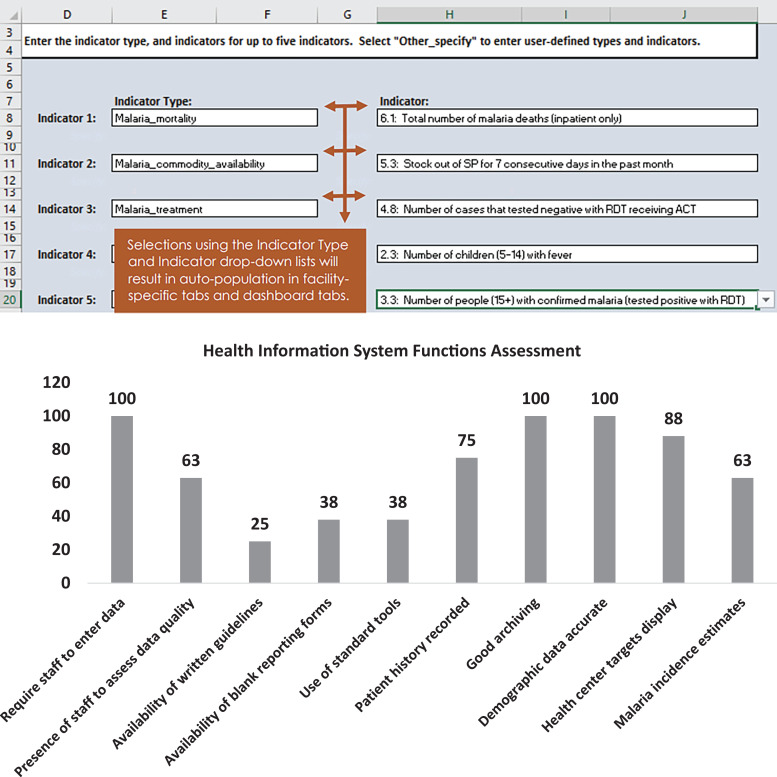
Screen shot and outputs, malaria routine data quality assessment tool (MRDQA) tool. Source: Measure Evaluation, MRDQA tool, 2020.

The MRDQA tool is an interactive Microsoft Excel form designed for assessing routine malaria data during regular supportive supervision of health facilities. It includes a comprehensive list of key malaria indicators that can be customized to prioritize country-specific indicators. By selecting the desired indicators, the tool generates comparative graphs or tables for each health center, displaying the outputs in a clear and concise manner. Furthermore, the tool provides a recommendations and actions table, outlining specific steps to address any identified gaps in the quality of data. Figure 2 shows a screen shot of the MRDQA tool used at health centers level.

### Selected program indicators.

The key program indicators assessed at health centers included 1) number of fever cases tested among 6- to 13-year-olds, 2) number of confirmed malaria cases among 6- to 13-year-olds, 3) number of confirmed malaria cases treated with artemisinin-based combination therapy (ACTs), 4) number of rapid diagnostic tests used, and 5) number of pregnant women who received at least three consecutive doses of treatment (sulfadoxine-pyrimethamine).

The age group 6- to 13 years was selected because malaria is prevalent in this population, which has a higher positivity rate compared with the other age groups.

### Data quality assessment metrics.

To ensure data quality adhered to specific standards, the following metrics from the MRDQA manual were used:

- ***Verification factor (VF):*** VFs are used to check the accuracy of selected indicators by comparing validated values for a defined reporting period with the reported values. In this assessment, malaria cases reported in monthly reports submitted to the health district were cross-checked with the cases reported in health center registers (source document). The VF is calculated by dividing the recounted (validated) value from the registers by the reported value (from the monthly report or DHIS2). Any VF value below 0.9 (90%) or above 1.1 (110%) indicates potential data quality issues, such as underreporting or overreporting, which should be investigated.
- ***Consistency ratio:*** the consistency ratio compares current indicator values with historical values in the same reporting period. In this assessment, the indicator values from the current month were compared with the same month of the previous year. Additionally, the current month’s value was compared with the average of the preceding 3 months. The annual consistency ratio is measured to identify any significant changes, except in cases of major demographic shifts in the facility’s catchment area. A difference of >20% (a ratio between 1.2 and 0.8) may indicate a data quality problem and warrants investigation. The **monthly consistency ratio** measures the consistency of values from month to month. A difference of 20% or more between the current month’s value and the average of the preceding 3 months (a ratio of <0.8 or >1.2) suggests potential data quality issues.
- ***Heath information system assessment:*** the health information system assessment focused on reviewing the tools and human resources necessary to ensure the system’s functionality. Nine items were assessed either by their presence (yes) or absence (no) at the health facility.

## RESULTS

### Characteristics of health centers assessed.

The health centers assessed were selected from settings with high malaria transmission (average annual malaria parasite incidence rate [API] over 100 cases per 1,000 inhabitants) and moderate malaria transmission (API rate between 50 and 100 cases per 1,000 inhabitants). Table 1 displays the distribution of health facilities based on transmission settings.

**Table 1:** Health centers distribution by region

Health Region	No. of Health Centers	Epidemiological Profile	Malaria Parasitic Incidence
Atsinanana	14	Moderate malaria transmission setting	The average annual malaria parasite incidence rate comprised between 50 to 100 cases per 1,000 inhabitants.
Atsimo Andrefana	16	High malaria transmission setting	Average annual malaria parasite incidence rate is >100 cases per 1,000 inhabitants.

Source: National Malaria Plan, strategic plan 2018–2023.

### Completeness of malaria monthly report.

Completeness of reporting consists of two indicators: the number of monthly summary reports submitted to the district compared with the expected number of summary reports. The adequate completeness performance as defined by NMP is 90% and more, with an acceptable rate between 70% and 90% and <70% as inadequate; and the completeness of data elements within the submitted monthly summary reports, which measures the number of data elements with missing data. Although monthly report submission completeness was 100% in each region, the assessment of the most recent completed and submitted malaria monthly facility report revealed that data element completeness (i.e., absence of unfilled or blank cells) was 43% for Atsinanana and 68% for Atsimo-Andrefana. However, data element completeness varied across health centers, ranging from 52% to 100%.

### Timeliness of submission of malaria monthly report.

In the previous 3 months leading up to the survey, the Atsinanana Health Centers had an average reporting timeliness rate of 85% for monthly reports submitted by the 15th of the following month to the district level. In contrast, the Atsimo Andrefana Health Centers had an average reporting timeliness of 95% for the same period.

The adequate timeliness performance as defined by NMP is 90% and more, with an acceptable rate between 70% and 90% and <70% as inadequate;

### Source documents completeness.

The source documents refer to the primary reporting documents used at the health center to record patient information and services provided, such as the outpatient register, monthly summary report, laboratory register, pharmacy dispensing log, ACT stock management log, long-lasting insecticide-treated net management log, and the malaria surveillance report. Source document completeness measures the quality of recording and determines the proportion of documents that are adequately filled without any missing data. In the assessment, it was found that the overall source document completeness was 72% for Atsinanana Health Centers and 92% for Atsimo-Andrefana Health Centers. However, the availability of guidelines to assist health workers in accurately filling out the source documents varied across different health centers, ranging from 7% to 14%.

### Reporting accuracy assessment.

To assess the accuracy of each health center, a thorough review was conducted on malaria case registers, HMIS monthly reports, and data extracted from DHIS2 for the previous 3 months. These sources were cross-checked, and VFs were estimated to identify any deviations from the normal VF range, which is defined as 0.90 = < VF < = 1.10. The selected health centers in Atsinanana had abnormal VF ranges for fever cases tested in children aged 6 to 13 years. Their VFs were <0.82 and >1.20. Similarly, in Atsimo-Andrefana, there were abnormal VF ranges for confirmed malaria cases treated with ACTs, with VFs exceeding 1.8 and 1.14.

**Figure 3.**
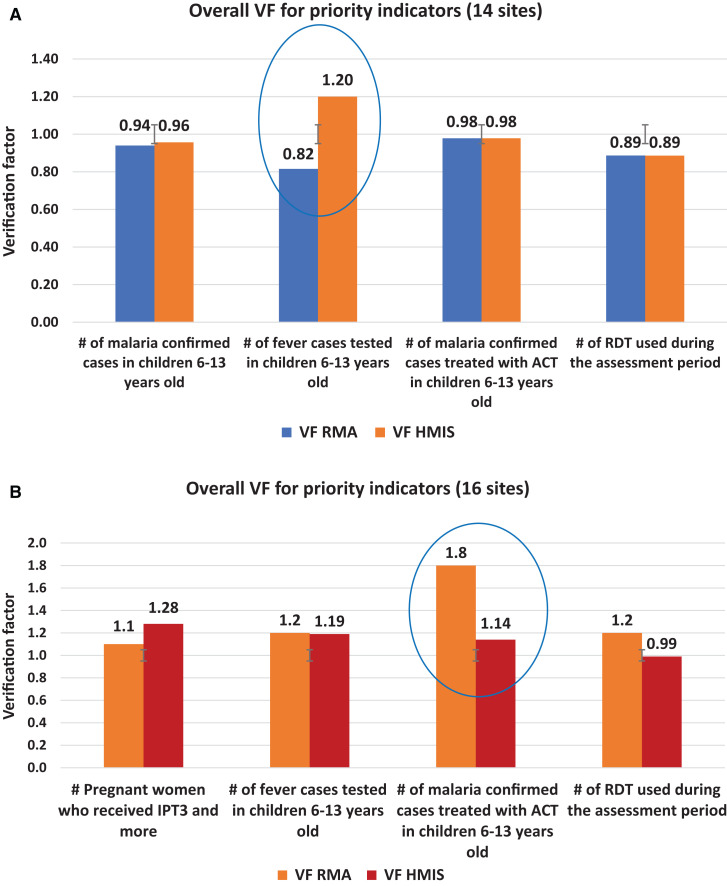
(**A**) Data accuracy measured by verification factor in Atsinanana health region. (**B**) Data accuracy measured by verification factor in Atsimo-Andrefana health region.

Figure 3 shows the comparative reporting accuracy among the two health regions:

### Data consistency over time.

To assess data consistency over time, month-to-month and annual consistency ratios were calculated for both health regions. The results revealed that five out of 14 health centers in Atsinanana (36%) and 8 out of 16 health centers in Atsimo-Andrefana (50%) fell outside the accepted normal range of 0.5–1.5.

**Figure 4.**
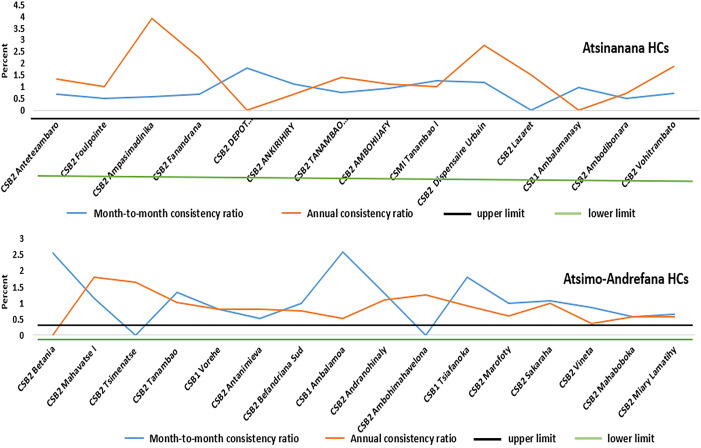
Month-to-month and annual consistency ratios in two health region.

Figure 4 provides a visual representation of the consistency ratios in the two health regions.

### Health information system assessment.

Both Atsinanana and Atsimo-Andrefana regions scored more than 80% for staff availability to enter data, compile reports, review data quality before submission at a higher level, use of standard registers to record malaria information, and availability of accurate demographic information for the catchment area. However, certain components recorded lower scores, including availability of written guidelines on data collection and reporting for malaria (14% in Atsinanana and 7% in Atsimo-Andrefana), reserve stock of registers or reporting forms (43% in Atsinanana and 64% in Atsimo-Andrefana), and established targets for health facility (57% in Atsinanana and 64% in Atsimo-Andrefana). Additionally, both regions were missing the chart of malaria incidence posted on wall.

**Figure 5.**
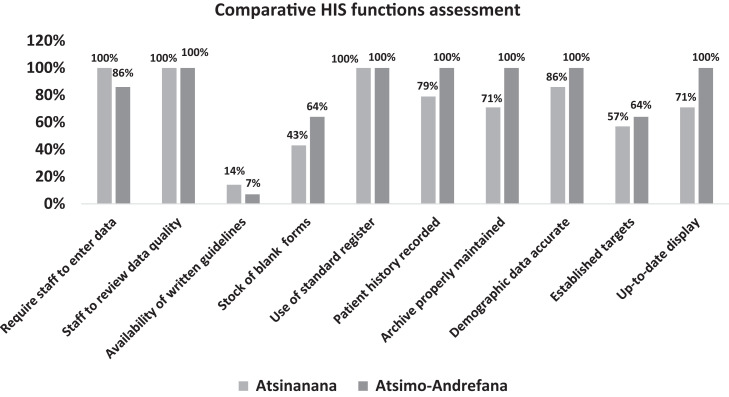
Comparative system assessment across the two health regions.

Figure 5 illustrates the assessment of the performance of the health information system by key components.

## DISCUSSION

The results of this study provide valuable insights into the characteristics of the health centers assessed and the data quality dimensions associated with malaria reporting and management in the two regions classified as moderate to high transmission settings. Our findings shed light on the completeness and timeliness of reporting, completeness of malaria data elements in source documents, accuracy of data reporting, and data consistency over time. Moreover, we assessed the availability of staff, tools, and resources required for quality health information services and the utilization of information for decision-making purposes.

Overall, our results indicate that the health centers assessed are representative of the malaria data collection, reporting, and management landscape in the selected regions. However, there are certain areas that require attention and improvement. For instance, the completeness of malaria data elements in monthly reports submitted to higher levels was relatively low in both regions, with proportions of 43% in Atsinanana and 68% in Atsimo-Andrefana. However, it is worth noting that all health centers assessed during the study period achieved a high performance of 100% completeness in monthly report submissions, with proportions of 85% in Atsinanana and 96% in Atsimo-Andrefana for timeliness.

The regional variation of data elements completeness performance indicates a potential issue with data reporting and documentation. This could mean that the health centers are not accurately and thoroughly capturing the required data elements in their reports. Inadequate understanding of reporting guidelines or lack of training in the use of reporting guideline for new staff or health workers shortage or overload can hamper their ability to collect and report data accurately and completely.

The completeness of source documents, such as registers and stock forms, was lower in Atsinanana (72%) compared with Atsimo-Andrefana (92%). Efforts are underway in Atsinanana to reach the national objective of achieving 90% completeness. These results are consistent with previous studies conducted in countries such as Senegal,^6^ the DRC (K. Johanna, personal communication, 2022), and Sierra Leone (M. Stanley, personal communication, 2022), suggesting that routine malaria data reporting has substantially improved over the years in these countries. The implementation of DHIS2 as a platform for data reporting and analysis may explain the high completeness and timeliness observed in our study and in Sierra Leone.

The accuracy of reporting raised some concerns, as we observed deviations from the normal range for key indicators in both regions. Specifically, the values for fever cases tested in children aged 6 to 13 years in Atsinanana and confirmed malaria cases treated with ACTs in Atsimo-Andrefana fell outside the normal range (0.90 = < = 1.10). Furthermore, data consistency over time was outside the norm (0.5–1.5) for a significant number of health centers in both regions, with rates of 36% in Atsinanana and 50% in Atsimo-Andrefana. This variability of month-to-month and annual consistency ratio across health centers who deviates significantly from the expected or normal range, could signify inconsistency in data reporting by health workers within registers or source documents, absence of clear guidelines for reporting and lack of training in health information system reporting tool. This could be exacerbated by staff high turnover experienced in most health facilities.

Similar findings have been reported in previous studies conducted in Senegal,^6^ Rwanda,^7^ Mozambique,^8^ and Ethiopia,^9^ suggesting that data quality issues are prevalent in malaria reporting systems across different countries.^10^ These studies highlight discrepancies in data accuracy, completeness, and timeliness, as well as the availability of written guidelines, standardized registers, and reporting forms. However, our study also acknowledges the progress made in some countries, such as the DRC (K. Johanna, personal communication, 2022), where health center data accuracy ratios were within the acceptable range.

On the basis of our findings, it is evident that improvements are needed in various aspects of the health information system to enhance the quality of routine malaria data for effective monitoring and decision-making. It is crucial to address issues related to data accuracy, availability, and completeness of source documents, as well as the provision of written guidelines and necessary resources to ensure the functionality of the health information system. Additionally, efforts should be made to promote consistent reporting over time to facilitate the monitoring and evaluation of malaria control and elimination efforts.

This study on malaria routine data quality assessment had limitations that reduce its generalizability countrywide. These include a nonrepresentative sample size of 30 health centers selected from two of the 22 health regions in Madagascar. Only high and moderate malaria transmission settings were chosen for the study, potentially weakening the understanding of malaria case management. Private health facilities were not included, limiting comparisons with government facilities. Other challenges such as staff turnover, insufficient supervision, and training gaps (although training is provided by PMI Measure Malaria project at district levels) also affected data quality. Additionally, the evaluation was considered a pilot study with limited trained staff and background data for the implementation of the MRDQA tool introduced in 2021.

In conclusion, our study highlights the importance of data quality in malaria reporting and management. It underscores the need for continuous efforts to improve the accuracy, completeness, and timeliness of routine malaria data and emphasizes the significance of a functional health information system for informed decision-making. The MRDQA tool provides an innovative solution for reviewing data consistently, identifying any gaps, and effectively addressing them through action plans developed with health center teams. To streamline the use of the tool, the PMI Measure Malaria project developed a mobile application version, specifically designed for Android devices.
